# Exploring the ethical issues in research using digital data collection strategies with minors: A scoping review

**DOI:** 10.1371/journal.pone.0237875

**Published:** 2020-08-27

**Authors:** Danica Facca, Maxwell J. Smith, Jacob Shelley, Daniel Lizotte, Lorie Donelle

**Affiliations:** 1 Faculty of Information and Media Studies, Western University, London, ON, Canada; 2 School of Health Studies, Western University, London, ON, Canada; 3 Department of Computer Science, Western University, London, ON, Canada; 4 Arthur Labatt Family School of Nursing, Western University, London, ON, Canada; Utrecht University Medical Center, NETHERLANDS

## Abstract

While emerging digital health technologies offer researchers new avenues to collect real-time data, little is known about current ethical dimensions, considerations, and challenges that are associated with conducting digital data collection in research with minors. As such, this paper reports the findings of a scoping review which explored existing literature to canvass current ethical issues that arise when using digital data collection in research with minors. Scholarly literature was searched using electronic academic databases for articles that provided explicit ethical analysis or presented empirical research that directly addressed ethical issues related to digital data collection used in research with minors. After screening 1,156 titles and abstracts, and reviewing 73 full-text articles, 20 articles were included in this review. Themes which emerged across the reviewed literature included: consent, data handling, minors’ data rights, observing behaviors that may result in risk of harm to participants or others, private versus public conceptualizations of data generated through social media, and gatekeeping. Our findings indicate a degree of uncertainty which invariably exists with regards to the ethics of research that involves minors and digital technology. The reviewed literature suggests that this uncertainty can often lead to the preclusion of minors from otherwise important lines of research inquiry. While uncertainty warrants ethical consideration, increased ethical scrutiny and restricting the conduct of such research raises its own ethical challenges. We conclude by discussing and recommending the ethical merits of co-producing ethical practice between researchers and minors as a mechanism to proceed with such research while addressing concerns around uncertainty.

## Introduction

Much has been written about the ethics of conducting research with minors, due in part to the distinctive ethical issues that emerge when conducting research with this population [1, 2]. Similarly, there is an emerging body of literature about the ethics of research practices that include digital data (sometimes characterized as ‘big data’) collection via digital technologies (e.g., smartphones). However, the ethical dimensions, considerations, and challenges that are associated with digital data collection in research involving minors remains unclear. Notably, does research that involves the generation and/or collection of digital data among minors present unique ethical challenges? What are those challenges and how might researchers best manage or mitigate the ethical dimensions and challenges within a digital technology context? This scoping review explores existing literature to understand and anticipate the ethical issues associated with collecting digitally derived research data with minors in addition to possible resolutions that can be put forward based on the reviewed literature.

### Minors and digital data

One challenge worth noting at the outset of this review is that there is no consensus as to the definition of a minor; two broad approaches could therefore be adopted for present purposes [3, 4]. The first defines a minor as any child who has not reached the age of majority, generally thought to be under the age of 18. The problem with this definition is that the age of majority varies by jurisdiction. Two notable documents which have been extremely influential in research ethics to the extent that many countries base their regulations on their guidelines, the *Declaration of Helsinki* [5] and the *International Ethical Guidelines for Health-related Research Involving Humans* [6], discuss age of majority and minors’ participation in research.

For example, within the North American context, the age of majority is 18 in some Canadian provinces like Ontario, Alberta, and New Brunswick, and 19 in other Canadian provinces like British Columbia, Nova Scotia, and the territory of Nunavut. Similar to Canada, in the United States, the age of majority also varies by state. In some U.S. states like Colorado, Idaho, and Minnesota the age of majority is 18, in others like Alabama and Nebraska it is 19, and in Mississippi it is 21. Within a European context, the age of majority in all EU Member States is 18 except for Scotland where it is 16 [7]. Additionally, the age of majority may depend on context. For example, in the Canadian province of Ontario, the age of majority is 18, but Ontario legislation dictates that one has to be 19 to purchase alcohol, suggesting that, in the context of alcohol purchases, an 18-year-old is a “minor”. Further, in some EU Member States, a minor will gain full legal capacity if they are married or become pregnant before reaching the age of majority [7], suggesting that, in the context of a marital contract or pregnancy, a minor can become an adult with full legal capacity even if they are under 18.

The second approach to defining a minor is based on capacity. This is consistent with established ethical guidelines which build on the *Declaration of Helsinki* [5] and the *International Ethical Guidelines for Health-related Research Involving Humans* [6] such as the *Tri-Council Policy Statement*: *Ethical Conduct for Research Involving Humans* [8]. According to the *Tri-Council Policy Statement*: *Ethical Conduct for Research Involving Humans*, if a child is “mature sufficiently to decide on their own behalf (subject to legal requirements), the researcher must seek the children’s autonomous consent in order for their participation to continue” [8]. For our purposes, it is not necessary that we resolve this issue, as our interest is in how others have addressed the issue of digital data collection in research with minors. As identified in Table 1 below, we adopted a broad approach, using keywords in our search intended as over-inclusive.

**Table 1:** Keywords for the academic literature search.

Ethical issues	Digital Data Collection	Research	Minors
ethic*	digital*	research*	kid*
moral*	electronic*		minor*
	online		adolescen*
	biosensor		teen*
	drone		millennial*
	device		child*
	“social media”		infant*

A second challenge worth noting is specifying what exactly is meant by “digital data”. For our purposes, while there may be overlap with what is otherwise termed ‘big data’—defined by the volume, variety, complexity, speed and value of the data—we broadly define digital data as electronic data or information however collected [9]. As Lupton notes: “People’s interactions online, their use of mobile and wearable devices and other ‘smart’ objects and their movements in sensor-embedded spaces all generate multiple and constant flows of digital data, often about intensely personal actions and preferences, social relationships and bodily functions and movements” [10]. It is in this data that our review is primarily interested.

## Methods

A multi-disciplinary research team was established to undertake this scoping review, with expertise in computer science, digital health, ethics, law, and public health. The scoping review was conducted according to Arksey and O’Malley’s [11] five stage framework which included: 1) identifying the research question/s; 2) identifying relevant studies; 3) selecting relevant studies; 4) charting the data; 5) aggregating, summarizing, and reporting the results. The research team consulted a research librarian who refined the initial search strategy and recommended databases to search given the context, subject, and population of interest.

### Literature search

To address the research question, scholarly literature was searched using four categories of keywords: *ethical issues*, *digital data collection*, *research*, *and minors* (Table 1). While initially considered, the search term ‘internet’ was purposefully excluded given the high volume of irrelevant articles captured in the search; the term ‘internet’ is so ubiquitous that it was not helpful to discriminate articles that addressed the purpose of the scoping review. Boolean search operators “AND” and “OR” were used to combine keywords between and within categories. Searches were conducted in PubMed and Scopus databases.

Articles were eligible for inclusion if they: were written in English; discussed the context of interest (i.e., research), subject of interest (i.e., digital data collection), population of interest (i.e., minors), and provided an explicit ethical analysis or presented empirical research that directly addressed ethical issues arising from the topic of interest (i.e., using digital data collection in research with minors). Upon completion of the search, duplicate articles were removed, and search results were screened by title and abstract for eligibility. Screening of abstracts was undertaken by a primary reviewer and confirmed by a second reviewer. Articles which passed the abstract screening were then retrieved in full-text and further screened for eligibility by a secondary reviewer and confirmed by two additional reviewers. Articles were summarized, and characteristics were charted, including: author(s), year of publication, database source, and ethical considerations. Reference lists of full-text articles were searched and eligible articles charted. Articles were thematically coded through multiple iterative discussions among three reviewers. A narrative account of key thematic findings is presented according to each identified theme.

### Search strategy

**Fig 1:**
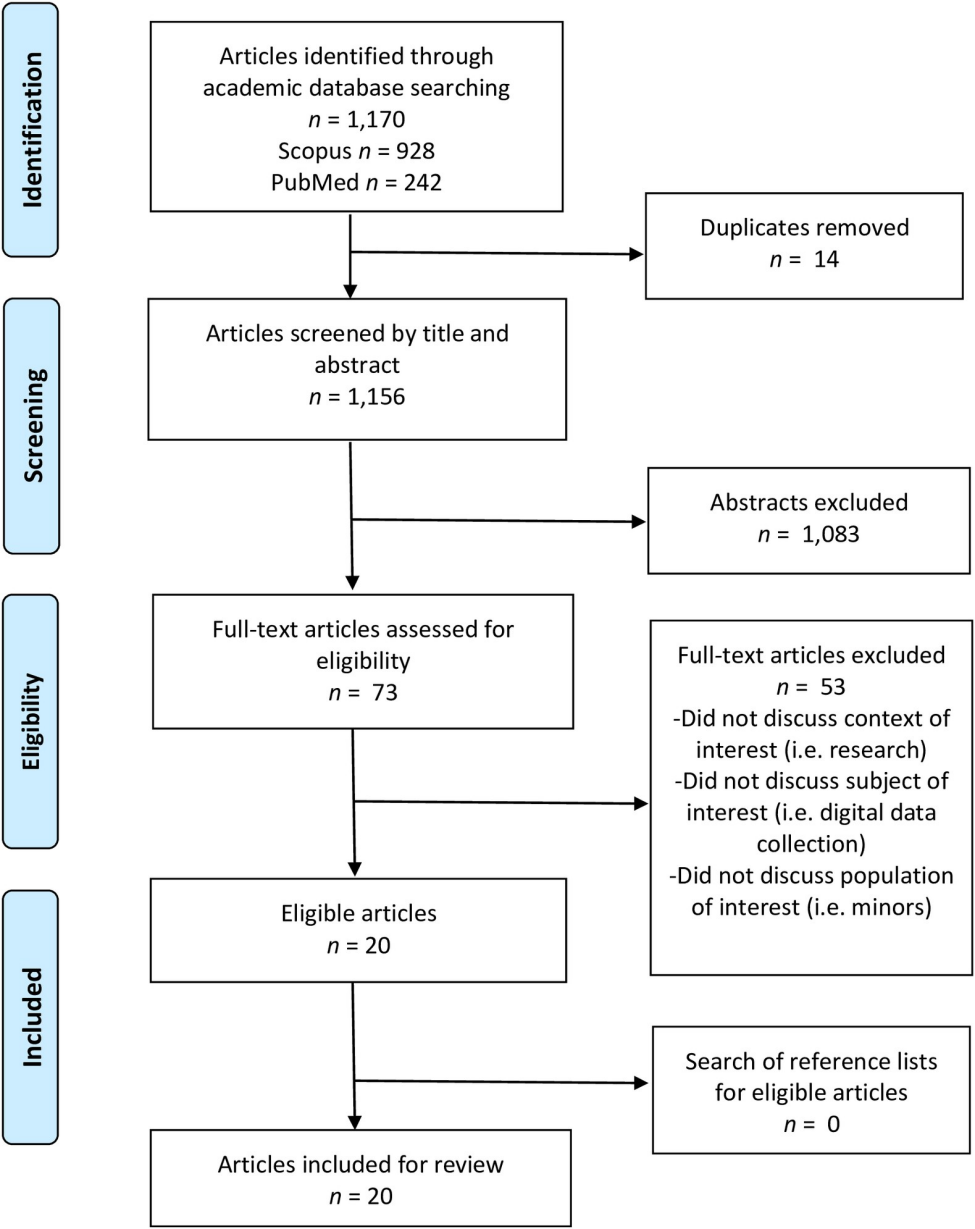
Search strategy and results.

The search strategy and results are summarized (Fig 1). Searching academic databases identified 1,170 abstracts, with 1,156 remaining following the removal of duplicates. Preliminary screening of abstracts identified 73 articles for full-text screening, of which 20 articles met the inclusion criteria. Hand searching reference lists did not identify any additional relevant articles, resulting in a total of 20 articles for inclusion in the scoping review.

### Scholarly literature characteristics

The majority of articles included in the review were published after 2014 (*n* = 14, 70%) and originated from the USA (*n* = 6, 30%), Australia (*n* = 6, 30%), and the UK (*n* = 3, 15%). More than half were original research (*n* = 11, 55%), including five qualitative studies (5/11, 45%). Overall, half of the articles (*n* = 10, 50%) focused on general ethical concerns with web- or internet-based digital data collection methods. Five articles (*n* = 5, 25%) focused specifically on the ethical concerns of using social media as a means of digital data collection. In general, social media (*n* = 9, 45%) was the most popular means of digital data collection. Facebook (*n* = 8/9, 88%) was the most popular platform identified, followed by Myspace (*n* = 3/9, 33%), YouTube (*n* = 2/9, 22%), Skype (*n* = 1/9, 11%), and Twitter (*n* = 1/9, 11%). Touch-screen technology was the second most popular means of digital data collection identified (*n* = 6, 30%) and included smartphones (*n* = 5/6, 83%) and tablets (*n* = 3/6, 50%). The remainder of digital data collection means included online forums (*n* = 4, 20%), computers (*n* = 2, 10%), internet-based surveys (*n* = 2, 10%), and an electronic health record system (*n* = 1, 5%). Of the participant samples described in the original research articles (*n* = 5), the majority age range of minors was 14 years of age and under (*n* = 3). One study focused on minors between 14 and 17 years of age, while another study focused on minors between 7 and 17 years of age (Table 2).

**Table 2:** Description of scoping review scholarly articles.

First author (Year)	Geographical location	Type of Scholarly Article	Research focus	Minor age range	Means of digital data collection
**Almeida (2017)**	Portugal	Original research (qualitative)	Minors’ use of digital devices at home and school	9–14	Desktop computer
**Cleminson (2015)**	United States	Report	Use of social media in research with pediatric populations	n/a	Social media (Facebook, YouTube, Skype)
**Cowie (2017)**	New Zealand	Original research	Ethical framework for conducting research with minors and ICTs	3.5–14	iPads, smartphone
**Denissen (2010)**	Germany, Sweden	Review	Psychosocial consequences of minors’ internet use	n/a	Internet-based questionnaires
**Donkin (2016)**	Australia	Original research (qualitative)	Minors’ use of virtual worlds/games (e.g., Minecraft)	5–12	Computer, laptop, iPad
**Eagleson (2017)**	Canada	Original research (intervention)	Impact of mobile lifestyle intervention in overweight and obese minors with congenital heart disease	7–17	Smartphone
**Fry (2014)**	Australia	Original research (qualitative)	Key informant views on the ethical concerns of Australia’s new PCHER system around minors	n/a	Personally controlled electronic health record (PCEHR) system
**Germain (2018)**	United Kingdom	Review	Uses, values, benefits, and ethical concerns of online research methods with minors and adults	13–18	Online forums, social media (Facebook, YouTube)
**Henderson (2012)**	United Kingdom	Case study	Ethical issues in pediatric pain research with minors	n/a	Online message board, internet-based therapy intervention
**Henderson (2013)**	Australia	Review	Researchers’ strategies used to handle ethical issues with research involving minors and social media	0–18	Social media
**Hokke (2018)**	Australia	Review	Ethical issues in engaging parents and minors in research online	0–18	Email, websites, online forums, social media (Facebook, Myspace)
**Kia-Keating (2017)**	United States	Original research (qualitative)	Ethical considerations for social media use in community-based participatory research with minors	n/a	Smartphones, social media
**Lunnay (2015)**	Australia	Original research (qualitative)	Social influences of drinking alcohol among minors	14–17	Social media (Facebook)
**Martin-Ruiz (2018)**	Spain	Methodologies	Ethical implications of minors’ data protection rights with ‘smart toys’	2–3	Smart toys (with accelerometers, pressure sensors, position sensors, etc.)
**Moreno (2008)**	United States	Original research	Ethical concerns when using social media for research purposes	n/a	Social media (Myspace, Facebook)
**Parsons (2015)**	United Kingdom	Original research	Potential of digital technologies to support minors’ rights and decision-making in research participation	n/a	Touch screen technology (tablet, smartphone)
**Schwab-Reese (2018)**	United States	Review	Use of social media and internet-based technologies for maltreatment research	n/a	Online surveys, online forums, social media (Twitter, Facebook)
**Shapiro (2013)**	United States	Report	Ethical and regulatory challenges of researching online educational games	n/a	Social media (Facebook)
**Spriggs (2009)**	Australia	Original research	Ethical issues of minors’ consent in cyber research	n/a	Social media (Myspace)
**Standlee (2017)**	United States	Methodologies	Ethical concerns of conducting digital ethnography with minors	n/a	Smartphones, social media (Facebook), blogs, online games

## Results

The thematic analysis generated 6 themes that addressed ethical considerations regarding digital data collection in research with minors which included: consent, data handling, minors’ data rights, observing behaviors that may result in risk of harm to participants or others, private versus public conceptualizations of data generated with social media, and gatekeeping. Although these themes can generally be applied to any research that leverages digital data collection, two out of the six themes, minors’ data rights and gatekeeping, warrant greater attention as they uniquely apply to conducting research when the population of interest is minors. The following sections detail the ethical issues raised according to each theme. For purposes of this review, the term *minors* is used interchangeably with any age descriptor identified in the reviewed literature (i.e., child/ren, kid/s, teen/s, adolescent/s, infant/s).

### Consent

Consent is an ethical requirement when conducting research with human participants. General principles of consent under policies that inform research practices like the *Tri-Council Policy Statement*: *Ethical Conduct for Research Involving Humans* [8], mandate that an individual’s consent must be given voluntarily and can be withdrawn at any time along with their data or biological materials upon request should they choose to withdraw their participation. To make such a decision, policies like the *Tri-Council Policy Statement*: *Ethical Conduct for Research Involving Humans* recommend that consent be determined by an individual’s decision-making capacity, that is, their ability “to understand relevant information presented (e.g., purpose of the research, foreseeable risks, and potential benefits), and to appreciate the potential consequences of any decision they make based upon this information” [8], rather than relying on age.

Autonomy, or the capacity needed to appreciate and understand the relevant information presented about a study in order to make an informed voluntary decision [8], is a necessary condition for consent. When it comes to involving minors in research, consent can be achieved in one of two ways: the researcher/s may obtain consent from the minor if it is determined that they have the capacity to make such an autonomous decision. Alternatively, the researcher/s may obtain consent from an authorized third party, like a parent or guardian, if it is determined that the minor does not have the capacity to make such an autonomous decision. If consent is obtained by an authorized third party, then assent to participate in the proposed research is sought from the minor. Consent by an authorized third party and assent are both needed for participation in research where it has been determined that a minor lacks autonomous decision-making capacity [8].

Numerous articles included in this review highlighted consent and assent as an ethical imperative when using digital data collection in research with minors [12–18]. Given the various geographical contexts of the research literature, the legal and ethical guidelines which determined the authors’ approaches to using digital data collection with minors varied respectively. Parsons [17] drew attention to digital data collection using digital technologies and the implications of this for participant recruitment and obtaining participant consent. Parsons [17] argued that digital technologies have the potential to support prospective participants’ autonomy, engagement, and decision making by: improving the accessibility of the research information; increasing motivation to take part in the study; and enhancing competency to inform decision-making. The author [17] further mentioned that digital technologies could be leveraged to make participant recruitment more inclusive, especially for minors with physical or learning disabilities. For example, using a laptop to present written text to prospective participants could give researchers the ability to tailor font size and color, background color, symbols, and computer-generated speech which can be paused, played, or slowed down, according to a minor’s needs [17]. Touch interfaces, like a tablet, were also a way to enhance recruitment and consent possibilities as they tended to be intuitive to users and did “not add unnecessary complexity to the learning process” [17].

Cowie and Khoo [12] approached issues of consent when using digital data collection in research with minors whereby minors are recognized as social actors and experts in their own lives. To this end, they informed prospective minor participants of the research aims and processes using multiple strategies to support minors’ decision to consent or dissent to participation in the research. They sent home a general newsletter about the study; created an interactive website about the study and research team for minors and their parents to view and discuss at home; and asked the minors’ teachers to explain the research aims in school [12]. Cowie and Khoo emphasized that once consent or assent is obtained from a minor participant in any study, researchers should treat it as provisional, that is to say, “ongoing and dependent on researcher-researched and inter-participant relationships…built upon sensitivity, reciprocal trust and collaboration” [12]. Treating consent as provisional and ongoing necessitates that researchers assess participants’ voluntariness at each point of contact and remind them of their right to withdraw their participation at any time [12].

Researchers [12, 17] also advocated for the use of digital technologies to facilitate the consent process as they offer “possibilities for multimodal/multimedia communication that improves the accessibility of research information beyond the inclusion of different fonts, formatting styles and images” [12]. For example, the use of digital video clips to recruit, inform, and debrief minor participants in an interactive manner engaged participants more effectively than a paper printout [12]. When considering the integral role and ethical imperative of obtaining consent from minors, Cowie and Khoo emphasized that the “onus is on researchers to use appropriate methods to achieve…consent in a way that scaffolds children’s understanding and encourages and maintains their voluntary and positive participation” [12].

One scoping review conducted by Hokke et al. [19] which explored the ethical issues of digital data collection in research with minors (i.e., using the internet to recruit prospective participants for family and child research) also discussed the challenges of obtaining consent and assent. In their review of research, they concluded that minor consent and parental consent was more complex and ethically challenging when facilitated through online means, rather than face to face interaction [19]. Many of the studies reviewed by the authors [19] noted that obtaining consent through online means posed a risk that minors would fraudulently complete their parents’ online consent form. To circumvent this risk, they [19] contacted prospective participants online, and then obtained verbal consent over the phone to assess parents’ and minors’ understanding of the research aims, procedures, and risks using back-questioning techniques.

Depending on the topic of interest, Hokke et al. [19] noted that waiving parental consent might in fact be a methodological and ethical necessity. For example, two US studies waived parental consent out of ethical necessity [20, 21] to collect digital data from gay and bisexual minors as they were considered to be at risk if they had to disclose their sexual identity to their parents as part of the consent process. Further, where the law required parental consent for a minor’s participation in research, researchers identified this as a possible deterrent for minors’ participation as some minors may be reluctant to ask their parents for permission [19].

### Data handling

One ethical imperative about the use of digital data collection with minors that was consistent throughout the reviewed literature was the importance of safeguarding data [8]. Safeguarding practices mentioned by researchers in the current review included data encryption, storage location, and secure server technology [14, 15, 22–24].

Some researchers [25] stressed that the various ethical issues at stake when handling digitally derived data depended on whether the data was actively, or passively, collected. For Schwab-Reese et al. [25], active data collection methods closely aligned with traditional data collection methods as they consist of direct interaction with research participants, even if the interaction is facilitated through electronic means [25]. Active data collection methods required participants to actively engage in the data collection process; in direct conversation with the researcher, or in responding to survey questions. For example, a researcher may ask a series of questions in real-time through a social media app to which a participant can give an immediate response. Alternatively, passive data collection methods aligned with secondary data analysis as they do not require direct interaction with participants, but rather, aggregate and analyze large sets of existing data [25]. For example, a researcher may monitor the number of hours a participant spends on a certain app, like Twitter, and therefore does not need to be in direct contact with the participant to collect that data in real-time.

Since a researcher would have direct contact with participants when using active data collection methods, informed consent or assent processes follow suit as standard practice [25]; the participant would be made aware that whatever data they produced in that given research context would be given over to the researcher for analysis. Examples of active digital data collection methods include crowdsourcing (i.e., most commonly done through websites where an open call for participation is issued for users to complete a short survey), or, online recruitment (i.e., most commonly done through websites or social media). Using active digital data collection methods in research with minors may not pose any additive ethical issues beyond those mentioned earlier when considering consent/assent processes as well as the degree to which the data are safeguarded through encryption, server technology, and storage location.

However, passive data collection methods proved to be more ethically challenging for researchers [25] when considering users’ privacy expectations about their digital data. Since passive data collection methods, according to Schwab-Reese et al. [25], are predicated on analyzing existing data, this means researchers may not have to necessarily seek out consent or assent from users which could be considered ethically problematic (e.g., does the user consider their digital data on a social media platform, like Twitter, private or public information?). Methods of passive digital data collection include internet search queries (e.g., Google Search Trends, which regularly updates a database of aggregated internet queries), forum postings (e.g., as found on a website like Reddit, or social media platform like Facebook), or social media user activity (e.g., analyzing Twitter users’ tweets, likes, retweets, use of certain hashtags, etc.).

To address the ethical queries related to the use of passive digital data collection methods with minors, researchers consulted guidelines from the Association of Internet Researchers [26] that recommended consideration of the following: what constitutes ‘engaging with a human subject’; definitions of private and public space; and finally, tensions between regulatory and context specific ethical decision making [27].

Online forums and social media platforms offer vast research opportunities for publicly available and passively collected data. On the surface, research processes required for ethical research ‘engagement with a human subject’ would not seemingly apply to the collection of publicly available online data. However, Schwab-Reese et al. noted that while passive data collection studies typically dealt with “aggregated or de-identified data…recent research suggests de-identified datasets often contain sufficient personal information to potentially identify individuals” [25]. Current recommendations for the ethical practice of online and publicly available passive data collection may exempt researchers from the customary oversight of an institutional ethics review board or regulatory body. Instead, researchers are recommending the establishment of “an external advisory committee to critically process the potential harms, vulnerabilities, benefits, and so forth, even if the research does not directly engage with human subjects and thus, does not explicitly require intuitional ethics review” [25]. They suggest this may be best practice moving forward in order to deal with research that digitally collects “person-based data without direct human contact” [25].

Ethical research practices pertaining to defining public and private space with respect to digital data collection methods should “carefully consider the expectations of the individuals who are creating the data” [25]. Different from active digital data collection methods, where prospective participants would be informed as to how their digital data was to be used for research purposes, this may not necessarily be the case with passive digital data collection methods. For example, “the creator of a public blog or social media profile which is viewable by anyone may have a lower expectation of privacy than in a private forum that requires log-in by members” [25]. Therefore, if researchers seek to passively collect digital data from minor participants, a combined approach of both active and passive data collection methods may be appropriate to understand the privacy expectations of prospective participants (active) before using their preexisting data or monitoring their behavior (passive).

Lastly, Schwab-Reese et al. acknowledged that “regulations are often intended to encourage ethical research and practice, but when applied universally without consideration, regulations may inadvertently restrict important, necessary research” [25]. With the increase of digital derived data collection, Schwab-Reese et.al. concluded that “as technology-based research moves forward, it will be important to establish firm ethical boundaries for some clearly defined issues, while encouraging flexibility and situation-based ethical decision-making for ethically grey areas” [25]. Ethical decision making on a case-by-case basis appears to be the way forward when considering the multitude of grey areas that arise when involving minors in research that uses digital data collection, whether active or passive in nature.

Benefits of using digital data collection were reported as an efficient, low cost, environmentally friendly, and convenient way to engage with a diverse range of participants across geographical boundaries [28]. However, challenges of digital data collection methods included technical aspects like data storage (servers) and security (encryption) measures, particularly as they related to the collection of personal health information [23, 24, 28]. Two studies mentioned a law or regulation with respect to the handling of personal health information collected through digital means [23, 24]. One study provided an extensive discussion related to necessary actions taken to comply with Ontario’s Personal Health Information Protection Act [29] which safeguards the collection and use of personal health information [23]. Martin-Ruiz et al. [24] reported on the use of the Data Protection Impact Assessment [30], a European regulation which assesses a study’s potential risks to individual privacy [30]. In their report, the researchers addressed the six protection goals regarding the collection and use of participants’ personal data [24] which included elements such as data availability, integrity, confidentiality, unlinkability, transparency, and intervenability [30]. As exemplified in both of these studies, addressing the ethical issues associated with handling digital data took both regulatory bodies and context-specific (personal health information) ethical decision making into consideration.

#### Minors’ data rights

The extent to which minors are included in discussions regarding the dissemination of their digital data for research purposes, or the sharing of their digital data with adult actors such as their parents or guardians generated through research, was frequently flagged as an ethical issue by many [12, 13, 16, 18, 22, 24, 31]. The contemporary issue of minors’ (digital) data rights is part of a much larger rights movement influenced by the establishment of the United Nations Convention on the Rights of the Child in 1989 [13, 22]. Historically, minors have been framed as a marginal group, with little, if any, opportunity to weigh in on matters (like research) that affect their social worlds [22]. The establishment of the convention signaled “the deconstruction of the protectionist paradigm of childhood [and] assigned [minors] the right to be co-producers of science and involved in all stages of research on and with them” [22] and consequently led to the emergence of childhood studies as a discipline. Opportunities for minors to be consulted and included in decision making processes is recognized by childhood studies’ scholars as a way to advance minors’ agency and autonomy within the research landscape [13].

Another ethical consideration in relation to minors’ data rights included privacy expectations of their parents/guardians [18]. Cultural and legal practices tend to normalize parent/guardian access to minors’ data held within government, healthcare or educational institutions; as such, parents/guardians may expect to have access to minors’ data collected for research purposes [18]. Drawing attention to minors’ data rights acknowledges their right to privacy over their personal data, both in an online and offline context, along with the need to obtain their consent in order to share their data with third party adult actors like their parents/guardians [32].

### Observing behaviors that may result in risk of harm to participants or others

Three studies mentioned the ethical challenges of reporting risky behaviors, or potential behaviors that could result in risk, when using digital data collection in research with minors [15, 33, 34]. In general, conducting research with human participants “includes the risk of observing or being informed about behavior that is illegal, amoral, immoral, or otherwise illicit” [33], to which researchers have to determine a course of action. Using digital data collection methods could potentially involve collecting information about minors’ online activity, for instance on social media, which may fall outside of a research study’s objectives but nonetheless be considered as illegal, amoral, immoral, or otherwise illicit. Under ethical guidelines like the *Tri-Council Policy Statement*: *Ethical Conduct for Research Involving Humans* such instances are known as material incidental findings as “they are reasonably determined to have significant welfare implications for the participant or prospective participant” [8].

Researchers [15, 33] highlighted the ongoing tension between existing laws and research ethics when conducting social media related research with minors. One study recognized that observing and responding to behaviors through social media which may harm participants or others poses complex challenges for researchers as it may be hard to decipher the parties involved (i.e., perpetrators, victims), the nature of the activity, or the proper contact to report the activity to [33]. For example, if a minor participant disclosed to a researcher that they illegally downloaded music through a social media platform, the researcher would need to decide if they would notify the participant and report the illegal activity, even if it meant jeopardizing their trust [33]. Similarly, researchers utilized the social media platform Facebook to contact under-aged women in Australia to explore the social influences of drinking alcohol [15]. Ethical challenges emerged for researchers regarding their responsibility to report incidents of illegal, amoral, immoral, or otherwise illicit behavior (i.e., photographs of inappropriate behaviors such as nudity, illegal activity) captured via online data collection against the promise of information confidentiality. Potentially reportable data was captured despite researchers’ caution to participants that “all information provided to the researcher would be treated in the strictest confidence [unless] the researcher was legally obliged to disclose information related to illegal activity if requested by relevant authorities” [15].

The ethical tension for researchers arises ‘in-between’ the requirement to disclose reportable incidents while ensuring an ethical duty of participant confidentiality whereby information obtained from a participant will remain confidential within and beyond a study [8]. While a researcher’s promise of confidentiality is central to developing and maintaining trusting relationships with participants, it “must, at times, be balanced against competing ethical considerations or legal or professional requirements that call for disclosure of information obtained or created in a research context” [8]. Depending on the topic and objective of a study, “researchers are expected to be aware of ethical codes…or laws…that may require disclosure of information they obtain in a research context” [8]. Breaching confidentiality by reporting confidential information risks losing the trust built between researcher and participant, but in some cases, it may be necessary in order to serve the greater good by protecting “the health, life or safety of a participant or a third party, a community, or the general population” [8].

Digitally collected data highlights unique tensions for researchers to maintain the integrity of the research purpose, to honor the confidentiality of participant information, and to disclose reportable events. For example, researchers [34] had to weigh the costs of constraining participants’ photographic activity against their study’s goal of equity and empowerment. They concluded that it was important “to allow youth to depict the reality of the challenges that they or their community faced without necessary constraint” [34]. Navigating the risk of harm to participants and their community proved to be complex as the authors realized minors often took photos on their own mobile phones rather than the digital cameras provided to them and uploaded the photos to their personal social media accounts to be shared on those platforms [34].

### Private versus public conceptualizations of social media

A large part of the reviewed literature discussed the ongoing debate of whether social media is considered private or public domain when using digital data collection in research with minors [13, 15, 19, 25, 33–37]. Many articles drew attention to the shifting nature of privacy within digital spaces since “any information posted on the Internet [technically] enters a virtual public space” [15] even if it is posted with the intention of remaining private to certain audiences. Understandings of privacy depend on the context in which it is invoked as well as a user’s expectations within that context. Since there is not a clear distinction between what is considered public and private domain within the internet, and social media more generally, maintaining user privacy and limiting potential harm surfaced as key ethical challenges [15, 33, 35, 36].

When using social media for research purposes, one study recommended researchers educate and ensure that their participants understand the Terms of Service associated with the social media site being used for the research project as it may lead to instances of public disclosure [33]. Another study stated that it is the researcher’s responsibility to also learn the nuances of the site’s privacy settings in order to inform their participants of the ways in which their information will be handled and, to a certain extent jeopardized, since privacy settings are not always fail-safe [15]. It was also mentioned that researchers should consider the cultural norms of the participants creating the data as well as the norms surrounding the social media platform being utilized to gauge what privacy means within these specific contexts [25].

When considering the cultural norms of the participants involved in research with social media, two studies recognized that “digital natives” [19], in other words, minors who are born into digital environments and learn how to use digital technologies from a young age, may conceptualize privacy differently than adults. For example, one article queried whether minors disclose private information on social media “without understanding or considering the permanence or far-reaching nature of online content, and without intending for their information to be used by others” [19]. While another mentioned that minors may disclose their private information on social media out of “naivety or ignorance” [37] rather than an intended disregard for their privacy and personal information.

### Gatekeeping

By far the most acknowledged ethical issue pertaining to the use of digital data collection in research with minors was gatekeeping by a parent/caregiver [12, 17, 19, 37], relevant stakeholders like medical professionals or educators [14, 18, 31] and research ethics boards [23, 25, 33, 36, 38]. While the inherent power imbalance between minors and adult researchers within the research context necessitates the need for gatekeeping by research ethics boards [12, 14], it is important to note that “the power [parents have] as gatekeepers in the processes of…research participation…should not be under-estimated” [17]. Parents/guardians ultimately provide access to minor participants, and in some cases, consent for their participation.

Conducting research with minors, irrespective of data collection method, must account for the triadic nature of the prospective research relationship which consists of the researcher, minor, and gatekeeper. Ultimately, it is the gatekeeper who grants a researcher access to a minor’s world [22]. Depending on the gatekeeper’s relationship to the minor, whether they are a parent/guardian, educator, or REB member, a minor’s prospective participation in a study will hinge on whether it is found to be in their best interest by these gatekeepers, and whether these gatekeepers are willing to cooperate with a researcher to make the study happen. When it comes to making decisions within the research context between adults, researchers, and minors, one authors suggests that research “decision-making needs to be understood as part of a discussion or dialogue between young people, parents/caregivers and the researchers” [17].

## Discussion

The reviewed literature identified numerous ethical issues related to conducting digital data collection in research with minors which included: consent, data handling, minors’ data rights, observing behaviors that may result in risk of harm to participants or others, private versus public conceptualizations of social media, and gatekeeping. While these ethical issues are pertinent to any discussion of using digital data collection in research, whether they are specifically unique when conducting such research with minors as the population of interest requires deeper consideration.

Although consent arose as a common theme among many of the studies, it is not an ethical concern unique to research with minors. Consent is, at the very least, a minimum requirement for research participation. Even so, seeking assent is not a unique ethical practice for research with minors as its tenets of accountability, reciprocal trust, and consultation should be considered an ethical necessity for all populations involved in research. When using digital data collection in research with minors, specifically within the context of social media, researchers [33] highlighted the importance of maintaining an open dialogue, or as they called it, a *dialogic approach*, throughout the research process. Following a dialogic approach with minors when using social media as an avenue for digital data collection allows researchers to assess minors’ feelings about their posts to social media and understandings of how published research will “transform…their private information and interactions into public data” [33]. Maintaining a continual dialogue with minor participants reaffirms the notion of provisional consent and that minors’ participation and data can be withdrawn at any time upon request.

While data handling and ownership are not unique ethical challenges when conducting research, they do warrant greater attention in a digital context. Since digital data encryption, secure storage location, and access are all ethical challenges which equally apply to research with minor and adult populations, the main ethical concern with data handling in this review was the extent to which the digital environment creates concomitant opportunities for data breaches. As noted by some researchers, “the very nature of the internet introduces security and privacy issues, including potential privacy breaches through hacking and data corruption during transfer” [23]. Given that security risks within a digital context are ever-present, a top priority for researchers using digital data collection methods will be ensuring, as is possible, that no manipulation, in other words hacking, happens during the transmission, encryption, and storage of participants’ digital data [24].

Observing behaviors which assume risk, or may result in risk to participants or others, is a conventional part of conducting research with human participants. While the reviewed literature acknowledged the ethical complexity of navigating such reporting in relation to participants’ social media data, deciphering what the activity is, what parties are involved, and whether there is an authority that the activity is required to be reported to, are ethical issues which can arise in any study with any population. As mentioned by some researchers, employing digital data collection methods warrants “an ongoing dialogue amongst researchers and ethics committees and between researchers and participants around…dilemmas, as well as processes to resolve them” [33]. They noted that the “constant evolution of technologies (such as social media and search capabilities) and social practices to which they are put…means that researchers and ethics committees are not necessarily equipped to understand the consequence or implications of their research practice” [33]. Therefore, within each individual research context that employs social media as a means to collect participants’ digital data, it is important for researchers to understand the social media practices of their population and approach dilemmas by applying all of the perspectives from those involved [33].

Varied conceptualizations of whether content shared through social media falls within the public or private domain is an important ethical issue when examining digital data collection methods, however, it is not unique to conducting research with minors. Although some articles claimed that digital natives may be naive or ignorant of social media privacy settings and the implications of sharing private information within digital spaces, adult users face similar struggles. With the rapid development, uptake, and variety of social media platforms across the globe, nuances of handling one’s personal information online affects users of all ages, backgrounds, and digital literacy levels; this is especially so given the lengthy and complex jargon of Terms of Service agreements [33]. Researchers will not necessarily face more struggles when conducting research with minors using digital data collection methods, such as social media, since users of all ages may not understand the intricacies and shifting nature of online contexts to the same degree [39].

### Managing minors’ data rights

Although minors’ data rights seems to point toward a unique ethical issue when conducting research with minors using digital data collection, many of the issues raised can be extended to other populations. For instance, weighing in on the treatment and dissemination of personal data for research purposes can advance the agency and autonomy of any aged participant. This is especially so given that the figure of the child tends to be positioned as “exceptional…rather than part of the wider frame of rights and the digital” [32]. While there may not be unique ethical concerns, in that other populations also have their best interests dictated by others such as the old, poor, or disabled, one might argue that where minors might differ is in their potential to be involved with research decision-making [32].

### Addressing gatekeeping in research involving minors and digital technology

Institutional review boards, or research ethics boards (REBs), dictate the scope of any research project for they are most concerned with risk mitigation [18]. Although REBs seek to reduce potential harm to participants while preserving the potential benefits of a research endeavor, they “can be a problematic gatekeeper for researchers, especially for those who are seeking to conduct research in new and contentious areas like online spaces” [33]. Since there is an additive degree of uncertainty that invariably exists when considering research that involves minors as well as digital technology, REBs themselves “may not even be equipped to best guide ethical practices concerning these new areas of inquiry” [33].

Gatekeeping is a unique ethical issue concerning research with minors that involves digital data collection. Considering that minors’ use of digital technology in general raises ambivalence among adults, it is no surprise that minors’ participation in research with digital technologies faces hypervigilant gatekeeping. As one author writes, research “decision-making needs to be understood as part of a discussion or dialogue between young people, parents/caregivers and the researchers” [17]. Perhaps the ethical issue at stake is not the population or topic of interest per se, but rather, the way in which gatekeeping may impede research efforts that can provide opportunities for minors to inform and shape understandings of our uncharted digital environments.

## Limitations

This review has several limitations. First, although the search strategy intended to be inclusive with its terminology, there may be relevant articles that were not captured. For example, articles may have described the context, subject, and population of interest without using ‘ethic’ or ‘moral’ in their title or abstract. This limitation also extends to the hand searched reference lists as only titles were screened for inclusion. Second, the search strategy was limited to articles written in English so work which may have contributed to this ethical discussion that was written in another language was not included. Third, the search strategy did not focus on one type of digital data to be collected (e.g., health data, screen-time data) which, if refined, could have identified further ethical issues or gaps in the literature. However, this was intentionally left outside of the scoping review search strategy as we were primarily concerned with the ethical issues of using digital data collection in research with minors, rather than the type of data being collected through digital means.

## Conclusion

As indicated at the outset of this review, our intention was to explore existing literature to understand and anticipate the ethical issues associated with collecting digitally derived research data with minors in order to forward any possible resolutions based on the reviewed literature. The reviewed literature indicated that there was no difference in ethical issues when collecting digitally derived research data with minors in comparison to other populations except for gatekeeping. Gatekeeping is a unique ethical issue when collecting digitally derived research data with minors given that it is both a necessary safety measure to ensure minors are not taken advantage of within the research context *and* a potential barrier to minors’ participation since digital technology is a contentious area of research. For our purposes, this ethical conundrum begets the following question: if gatekeeping is a necessary barrier to minors’ participation in research which specifically involves the collection of digitally derived research data, how do we resolve this as researchers based on the reviewed literature? Our resolution to this conundrum is to suggest that researchers co-produce ethical practice with minors.

At various points throughout the reviewed literature, it was continually suggested that any ethical issue associated with digital data collection in research with minors may be best addressed when minors are part of the research conversation and decision-making processes [12,13, 16–18, 22, 24, 31, 33]. In this sense, co-producing ethical practice with minors is the most respectable resolution to approaching ethical dilemmas in an area of research where the technology itself, along with accompanying social practices, are constantly evolving. Co-producing ethical practice in research which collects digitally derived research data from minors could be addressed by implementing a Child and Youth Advisory Committee (CYAC) [37]. CYACs seek to balance children’s protection while supporting their participation in research [40]. In particular, CYACs have been implemented in research with minors which addressed similarly contentious topics including cyber safety [41], hazardous agricultural labor [42], self-advocacy for pediatric patients with chronic illness [43], and child rights [40].

The problem when using digital data collection in research with minors is not necessarily the minors, nor the digital technology, but the uncertainty surrounding it. Conducting research with minors, along with digital technology, compounds uncertainty and increases ethical scrutiny. Uncertainty should not lead to preclusion, but rather, to co-production of ethical practice between researchers and minors. Co-production *is* risk mitigation; it is not an antidote to risk but an approach to working in tandem with minors to foster best ethical practice when using digital means to collect their data within a research context.

## Supporting information

S1 File (PDF)

## References

[1] Bos W., Tromp K., Tibboel D., Pinxten W. Educational Paper: Ethical aspects of clinical research with minors. Eur J Pediatr.172:859–866. 10.1007/s00431-012-1856-8 23073901

[2] Fargas-Malet M., McSherry D., Larkin E., Robinson C. Research with children: methodological issues and innovative techniques. J Early Child Res.2010;8(2):175–192. 10.1177/1476718X09345412

[3] Campbell T.D. The rights of the minor: As person, as child, as juvenile, as future adult. Int J Law Fam.1992;6(1):1–23. 10.1057/cpt.2011.43

[4] Cowden M. Capacity, claims and children’s rights. CPT.2012;11:362–380. 10.1057/cpt.2011.43

[5] WMA Declaration of Helsinki–Ethical Principles for Medical Research Involving Human Subjects. World Medical Association. 2018 July. Available from: https://www.wma.net/policies-post/wma-declaration-of-helsinki-ethical-principles-for-medical-research-involving-human-subjects/19886379

[6] International Ethical Guidelines for Health-related Research Involving Humans, Fourth Edition Geneva Council for International Organizations of Medical Sciences (CIOMS); 2016 Available from: https://cioms.ch/wp-content/uploads/2017/01/WEB-CIOMS-EthicalGuidelines.pdf 40523065

[7] Mapping minimum age requirements concerning the rights of children in the EU. European Union Agency for Fundamental Rights. 2017 Nov. Available from: https://fra.europa.eu/en/publication/2017/mapping-minimum-age-requirements/age-majority

[8] Canadian Institutes of Health Research, Natural Sciences and Engineering Research Council of Canada, and Social Sciences and Humanities Research Council. Tri-Council Policy Statement: Ethical Conduct for Research Involving Humans. 2018 Dec. Catalogue No: RR4-2/2019E-PDF. ISBN: 978-0-660-29942-6.

[9] Hiba Jasmin H., Ammar Hameed S., Hadisheheed S., Azizahbt Haji A. Big Data and the Five V’s Characteristics. IJAECS.2015;2(1):16–23. 10.1007/978-3-319-06811-4_13

[10] Lupton D. Feeling your data: Touch and making sense of personal digital data. New Media Soc.2017;19(10):1599–1614. 10.1177/1461444817717515

[11] Arksey H., O’Malley L. Scoping studies: towards a methodological framework. Int J Soc Res Methodol.2005;8(1):19–32. 10.1080/1364557032000119616

[12] Cowie B., Khoo E. Accountability through access, authenticity and advocacy when researching with young children. Int J Inclu Edu.2017;21(3):234–247. 10.1080/13603116.2016.1260821

[13] Donkin A., Holloway D., Green L. Towards a participatory Netnography: collaborating with children in virtual worlds research. J Med Comm.2016;7:5–16. Available from: https://platformjmc.files.wordpress.com/2016/10/adonkin_netnography_fullpaper.pdf

[14] Henderson E.M., Law E.F., Palermo T.M., Eccleston C. Case Study: Ethical Guidance for Pediatric e-health Research Using Examples From Pain Research With Adolescents. J Pediatr Psychol.2012;37(10):1116–1126. 10.1093/jpepsy/jss085 22851643 PMC3529561

[15] Lunnay B., Borlagdon J., McNaughton D., Ward P. Ethical Use of Social Media to Facilitate Qualitative Research. Qual Health Res.2015;25(1):99–109. 10.1177/1049732314549031 25212856

[16] Moreno M.A., Fost N.C., Christakis C.D. Research Ethics in the Myspace Era. Pediatrics.2008;121(1):157–161. 10.1542/peds.2007-3015 18166570

[17] Parsons S. The Potential of Digital Technologies for Transforming Informed Consent Practices with Children and Young People in Social Research.JoSi.2015;3(6):56–68. 10.17645/si.v3i6.400

[18] Standlee A. Digital Ethnography and Youth Culture: Methodological Techniques and Ethical Dilemmas. Social Stud Child Youth.2017;22:325–348. 10.1108/S1537-466120180000022015

[19] Hokke S., Hackworth N.J., Quin N., Bennetts S.K., Win H.Y., Nicholson J.M. et al Ethical issues in using the internet to engage participants in family and child research: A scoping review. PLoS ONE.2018;13(9):1–30. 10.1371/journal.pone.0204572 PMC6160098 30261041

[20] Hoffman N.D., Freeman K., Swann S. Healthcare preferences of lesbian, gay, bisexual, transgender and questioning youth. J Adolesc Health.2009;45(3): 222–229. 10.1016/j.jadohealth.2009.01.009 19699417 PMC2773204

[21] Nielsen S., Paasonen S., Spisak S. ‘Pervy role-play and such’: girls’ experiences of sexual messaging online. Sex Educ.2015;15(5):472–85. 10.1080/14681811.2015.1048852

[22] De Almeida A.N., Carvalho D., Delicado A. Accessing Children’s Digital Practices At Home Through Visual Methods: Innovations and Challenges. Sociol Stud Child Youth.2017;22:349–374. 10.1108/S1537-466120180000022016

[23] Eagleson R., Altamirano-Diaz L., McInnis A., Welisch E., De Jesus S., Prapavessis H. et al Implementation of clinical research trials using web-based and mobile devices: challenges and solutions. BMC Med Res Methodol.2017;17(43):1–8. 10.1186/s12874-017-0324-6 28302050 PMC5356263

[24] Martin-Ruiz M.L., Fernandez-Aller C., Portillo E., Malagon J., del Barrio C. Developing a System for Processing Health Data of Children Using Digitalized Toys: Ethical and Privacy Concerns for the Internet of Things Paradigm. Sci Eng Ethics.2018;24(4):1057–1076. 10.1007/s11948-017-9951-x 28815460

[25] Schwab-Reese L.M., Hovdestad W., Tonmyr L., Fluke J. The potential use of social media and other internet-related data and communications for child maltreatment surveillance and epidemiological research: Scoping review and recommendations. Child Abuse Negl.2018;85:187–201. 10.1016/j.chiabu.2018.01.014 29366596 PMC7112406

[26] Buchanan E. Readings in Virtual Research Ethics: Issues and Controversies. Information Management.2005 Spring;18(1):26–27. Available from: https://www.researchgate.net/publication/293512050_Readings_in_virtual_research_ethics_Issues_and_controversies/stats

[27] Markham A., Buchanan E. Ethical decision-making and internet research: Recommendations from the AoIR ethics working committee (version 2.0).2012 Summer. Available from: http://aoir.org/reports/ethics2.pdf

[28] Denissen J.J.A., Neumann L., van Zalk M. How the internet is changing the implementation of traditional research methods, people’s daily lives, and the way in which developmental scientists conduct research. Int J Behav Dev.2010;34(6):564–575. 10.1177/0165025410383746

[29] Personal Health Information Protection Act, 2004, S.O. 2004, c. 3, Sched. A. (March 25, 2020). Available from: https://www.ontario.ca/laws/statute/04p03

[30] Data Protection Impact Assessment. (March 12, 2019). Available from: https://edpb.europa.eu/our-work-tools/our-documents/topic/data-protection-impact-assessment-dpia_en

[31] Fry C.L., Spriggs M., Arnold M., Pearce C. Unresolved Ethical Challenges for the Australian Personally Controlled Electronic Health Record (PCEHR) System: Key Informant Interview Findings. AJOB Empir Bioeth.2014;5(4):30–36, 10.1080/23294515.2014.919972

[32] Livingstone S., Third A. Children and young people’s rights in the digital age: An emerging agenda. New Media Soc.2017;19(5):657–670. 10.1177/1461444816686318

[33] Henderson M., Johnson N.F., Auld G. Silences of ethical practice: dilemmas for researchers using social media. Educ Res Eval.2013;19(6):546–560. 10.1080/13803611.2013.805656

[34] Kia-Keating M., Santacrose D., Liu S. Photography and Social Media Use in Community-Based Participatory Research with Youth: Ethical Considerations. Am J Community Psychol.2017;60:375–384. 10.1002/ajcp.12189 28944473 PMC5735042

[35] Germain J., Harris J., Mackay S., Maxwell C. Why Should We Use Online Research Methods? Four Doctoral Health Student Perspectives. Qual Health Research.2018;28(10):1650–1657. 10.1177/1049732317721698 28745106

[36] Shapiro R.B., Ossorio P.N. Regulation of Online Social Network Studies. Science.2013;339(6116):144–145. 10.1126/science.1219025 23307724

[37] Spriggs M. Consent in Cyberspace. Monash Bioeth Rev.2009;28(4):32.1–32.15. doi: 10.2104jmber0932 20440984

[38] Cleminson J. Child health research and social media. Arch Dis Child Educ Pract Ed.2015;100:331–332. 10.1136/archdischild-2014-308100 25824892

[39] Obar J.A., Oeldorf-Hirsch A. The biggest lie on the Internet: ignoring the privacy policies and terms of service policies of social networking services. Inf Comm Soc.2020;23(1):128–147. 10.1080/1369118X.2018.1486870

[40] Collins T.M., Jamieson L., Wright L.V.H., Rizzini I., Mayhew A., Narang J., et al Involving child and youth advisors in academic research about child participation: The Child and Youth Advisory Committees of the International and Canadian Child Rights Partnership. Child Youth Serv Rev.2020;109:1–9. Retrieved from: 10.1016/j.childyouth.2019.104569

[41] Lester L., Cross D., Terrelinck D., Falconer S., Thomas L. Encouraging the positive use of technology through community engagement. Safer Communities.2016;15(3):134–141. 10.1108/SC-11-2015-0035

[42] Arnold T.J., Malki A., Ibarra J., Daniel S.S., Ballard P.J., Sandberg J.C., et al Engaging Youth Advocates in Community-Based Participatory Research on Child Farmworker Health in North Carolina. PCHP.2019;13(2):191–199. Retrieved from: 10.1353/cpr.2019.0019 31178454 PMC6559374

[43] Rich C., Goncalves A., Guardiani M., O’Donnell E., Strzelecki J. Teen Advisory Committee: Lessons Learned by Adolescents, Facilitators, and Hospital Staff. Pediatr Nurs.2014;40(6):289–296. Retrieved from: https://pubmed.ncbi.nlm.gov/25929124/ 25929124

